# Stratification of Phenotypes in Childhood-Onset *COL4A1/COL4A2*–Related Disorders Based on Age of Presentation

**DOI:** 10.1212/NXG.0000000000200386

**Published:** 2026-05-04

**Authors:** Giulia S. Porcari, Rudmila N. Rashid, Caitlyn A. Mulvihill, Lauren A. Beslow, Holly A. Dubbs, Erica M. Schindewolf, Michael C. Kaufman, Alexander K. Gonzalez, Juliana S. Gebb, Sonika Agarwal, Matthew T. Whitehead, Ana G. Cristancho

**Affiliations:** 1 Division of Neurology, Children's Hospital of Philadelphia, PA;; 2 Departments of Neurology and Pediatrics, Perelman School of Medicine at the University of Pennsylvania, Philadelphia;; 3 Translational Research Informatics Group, Children's Hospital of Philadelphia, PA;; 4 Richard D. Wood Jr Center for Fetal Diagnosis & Treatment, Division of Pediatric General, Thoracic and Fetal Surgery, Department of Surgery, Children's Hospital of Philadelphia, PA; and; 5 Division of Neuroradiology, Department of Radiology, Children's Hospital of Philadelphia, PA.

## Abstract

**Background and Objectives:**

Variants in *COL4A1* and *COL4A2* are associated with a multisystem disorder characterized by prominent neurologic involvement that includes intracranial hemorrhages, white matter injury, neurodevelopmental impairment, and epilepsy. The phenotypic spectrum, however, is broad, and disease subgroups have not been robustly identified. The objective of this study was to distinguish pediatric subgroups based on age at symptom onset.

**Methods:**

This was a retrospective cohort study of pediatric patients with variants in *COL4A1* or *COL4A2* seen at a single center between January 2008 and October 2024. Patients were included if they had likely pathogenic/pathogenic variants or variants of uncertain significance with consistent clinical phenotype and were followed for ≥6 months. Medical records, laboratory data, and neuroimaging were reviewed. Patients were stratified by age at symptom onset into perinatal, early childhood, and late childhood onset (up to 28 days, up to 4 years, and up to 18 years, respectively).

**Results:**

Of the 44 patients meeting inclusion criteria, 33 had variants in *COL4A1*, 10 in *COL4A2*, and 1 in both. Neurologic features, such as global developmental delay, cerebral palsy, and epilepsy, were common in perinatal and early childhood cases. In *COL4A1*-related disease, such neurologic features were present in 14/17 and 8/9 cases, respectively. These features similarly occurred in all patients with perinatal (n = 3) and early childhood (n = 6) onset of *COL4A2*-related disease. Conversely, these manifestations were less common in late childhood presentations of either disorder (n = 6 total), occurring in 33% of patients. Extracentral nervous system manifestations, particularly ocular abnormalities and renal disease, were predominantly seen in *COL4A1*-related disease. Neuroimaging in perinatal and early childhood presentations frequently demonstrated periventricular hemorrhagic infarction (20/26 and 6/9 of *COL4A1* and *COL4A2* patients). Isolated leukoencephalopathy was universally present in late childhood onset patients.

**Discussion:**

The pediatric phenotype of *COL4A1/2*-related disorder varies by age at disease onset. Perinatal and early childhood presentations (≤4 years) have a prominent neurologic phenotype with severe developmental delays, cerebral palsy, and epilepsy, correlating on imaging with sequelae from brain injury during prenatal brain development. Late childhood presentations (>4 years) have a milder phenotype, typically with isolated leukoencephalopathy on imaging.

## Introduction

*COL4A1* and *COL4A2* encode the 2 most abundant subunits of type IV collagen, the main collagen in vascular endothelium. Variants in these genes, whose protein products have significant homology and form ⍺1⍺1⍺2 heterotrimers, have been associated with a phenotype of early-onset autosomal dominant intracerebral hemorrhages known as autosomal dominant familial porencephaly.^1,2^ Since their initial discovery, this core phenotype has been extended into a multisystem disorder, with several additional named syndromes proposed such as autosomal dominant brain small vessel disease with hemorrhage type 1, pontine autosomal dominant microangiopathy and leukoencephalopathy, and hereditary angiopathy and nephropathy with aneurysms and muscle cramps (HANAC).^1-20^ A spectrum of ocular manifestations, ranging from isolated retinal artery tortuosity to anterior segment dysgenesis, is also recognized.^20,21^ While efforts are ongoing to standardize clinical surveillance, there are limited data on the disease's natural history.^20,22^ This hampers counseling, clinical management, and, ultimately, efforts to develop targeted interventions, particularly in light of increased recognition of the disorder. Indeed, *COL4A1/2* variants have been identified in up to 28% of fetuses prenatally diagnosed with multifocal hemorrhagic lesions, with distinct implications for prenatal counseling.^23,24^

While the literature and anecdotal experience suggest an association between age at symptom onset and phenotype, this has not been systematically assessed. Notably, given the rarity of these disorders and differential access to genetic testing, there is often a lag between symptom onset and diagnosis. We hypothesized that age-based phenotypes can be identified, characterized by distinct burdens of neurologic and non-neurologic symptoms, and neuroimaging findings.

## Methods

### Patient Ascertainment

This was a retrospective cohort study of patients evaluated at the Children's Hospital of Philadelphia. Patients were identified through a regular expression query of electronic health records (EPIC, Verona, WI), stored in an institutional data warehouse. Records containing mentions of *COL4A1* or *COL4A2* between January 2008 and October 2024 were retrieved and manually reviewed by authors to confirm patients met genetic and age-based inclusion criteria. Genetic inclusion criteria included confirmed variants classified as pathogenic or likely pathogenic according to American College of Medical Genetics and Genomics criteria, or variants of uncertain significance with a consistent clinical phenotype and no alternative diagnosis. Clinical inclusion criteria included presentation to care <18 years and age at last follow-up of ≥6 months, allowing for sufficient time to observe early developmental course. Fetal patients not carried to term and patients with *COL4A1/2* copy number variants (CNVs) extending beyond genes of interest were not included.

### Data Abstraction and Neuroimaging Severity Scoring

Demographic information, family history, neurologic and extra-neurologic manifestations, surgical interventions, medications, laboratory, and imaging data were abstracted from medical records. We specifically assessed for the following signs or symptoms as presenting manifestations of disease: (1) neurologic disease: abnormalities on prenatal imaging (including but not limited to ventriculomegaly, intraventricular and intraparenchymal hemorrhage, porencephaly, schizencephaly, and white matter abnormalities), seizures, abnormal tone, developmental delay(s), cerebral palsy, autism, attention deficit hyperactivity disorder (ADHD), intellectual disability, and migraines; (2) ophthalmologic disease: anterior segment dysgenesis, cataracts, glaucoma, and retinal vasculature abnormalities; (3) renal disease: proteinuria, hematuria, elevated creatinine, and cystic malformations; (4) cardiac disease: any cardiac abnormalities aside from patent foramen ovale in the perinatal period; and (5) musculoskeletal disease: musculoskeletal pain, cramps, and elevated creatinine kinase. “Global developmental delay” was defined by the presence of this diagnosis in documentation or by reported delays in both motor and language domains. “Cerebral palsy” (CP) was defined as presence of this diagnosis in documentation or a persistent motor deficit apparent before 12 months of age. The Gross Motor Function Classification System (GMFCS), designed to capture motor function in children with CP, was retrospectively applied to the last available neurology note to estimate motor deficit severity.^25^ A composite outcome of “profound neurologic impairment” was defined as the presence of global developmental delay, CP with GMFCS of ≥4, refractory epilepsy, or death. Neurologic features not explicitly documented were considered absent; prevalence of extra-neurologic disease manifestations, conversely, was calculated only among patients in whom relevant screening was pursued given variable screening practices. Clinical data were reviewed by 2 abstractors (G.P. and R.R. or C.M.).

A pediatric neuroradiologist (M.W.) reviewed magnetic resonancce imaging (MRI). In an effort to quantify degree of brain injury, we adapted a severity score from the pediatric neuroradiologic literature, as no validated score exists for this population. This score was developed in newborns treated with extracorporeal membrane oxygenation, who, similarly to patients with *COL4A1/COL4A2* variants, exhibit neuroimaging patterns of complex injury with both hemorrhagic and ischemic infarctions in multiple brain areas.^26^ The score assesses the location and severity of parenchymal injury (hemorrhagic and ischemic), extra-axial hemorrhage, and enlargement of cerebrospinal fluid (CSF) spaces. Increasing scores reflect worse injury; scores of 0 correspond to none, 1–13.5 have been described as mild/moderate, and ≥14 as severe.

### Group Stratification

Patients were stratified as follows based on age at first disease-specific sign or symptom: perinatal (prenatal period up to 28 days of life), early childhood (>28 days of life up to 4 years), and late childhood/adolescence (>4 years up to 18 years of life). Cutoffs were chosen based on distribution of ages (eFigure 1) and thorough review of clinical course, neuroimaging, and outcomes, and informed by the goal of creating robust groups that would also be relevant to clinical practice.

### Statistical Analyses

Descriptive statistics are presented as counts and percentages for categorical variables, and medians with interquartile ranges (IQRs) for continuous variables. Fisher exact test was used to compare proportions of patients with profound neurologic impairment across genes and subgroups. Wilcoxon signed-rank test was used to compare median age at onset and diagnosis. Kruskal-Wallis test and Mann-Whitney *U* tests were used to compare imaging severity scores across subgroups. Logistic regression was used to assess association between profound neurologic impairment, age, and neuroimaging severity, limiting adjustment for confounders to sex and gene given sample size. The first postnatal scan was chosen for such analyses for consistency across patients, to limit risk of reverse causality fallacy, and as expected to be the most relevant to early clinical prognostication. A *p* value <0.05 was considered significant. All statistical analyses were completed in STATA (BE version 18.0, StataCorp College Station, TX).

### Standard Protocol Approvals, Registrations, and Patient Consents

This study was reviewed by the Children's Hospital of Philadelphia Institutional Review Board (IRB 22-020166) with a waiver of HIPAA authorization per 45 CFR 164.512(i)(2)(ii) to access identifiable information from medical records. Deidentified data were securely stored in Research Electronic Capture (REDCap).^27^

### Data Availability

Deidentified data not published are available on reasonable request from investigators.

## Results

### Overall Study Population Characteristics

Three hundred thirty-six unique patient charts were identified, with 60 meeting genetic inclusion criteria (eFigure 2). Reasons for exclusion included mention of testing without recommendation to test, recommended testing not completed, negative, or consistent with a VUS predicted to be benign/nonexplanatory or with a CNV extending beyond *COL4A1*/*COL4A2,* and miscoding. Seven cases were fetal not carried to term, and 10 were adult parents.

The final study population included 44 patients, 33 with variants in *COL4A1*, 10 in *COL4A2*, and 1 with variants in both genes. To our knowledge, none of these patients have been previously described. Demographic features are summarized in eTable 1. Among patients with variants in *COL4A1*, median age at presentation with disease-related signs or symptoms was 0.2 years (IQR 0.0–0.9) and was significantly earlier than median age at diagnosis of 1.4 years (IQR 0.2–8.6) (n = 31, *p* = 0.0001). Two patients diagnosed in the setting of family history at 1.1 and 14 years, respectively, remained asymptomatic during follow-up. Patients were followed for a median of 2.5 years (IQR 0.3–3.6). Two patients (2/33, 6%) died <2 years of age in the setting of respiratory failure, apnea, and worsening seizure burden and exacerbated in one by a febrile respiratory illness. Among patients with variants in *COL4A2*, median age at presentation and diagnosis were 0.5 years (IQR 0.0–1.3) and 2.8 years (IQR 0.9–4.4), respectively (n = 10, *p* = 0.06). Patients were followed for a median of 1.3 years (IQR 0.6–1.8). Overall, there was no difference in time from symptom onset to diagnosis by gene (*p* = 1.00), although diagnostic delay did decrease over time (eFigure 3).

### Phenotypes by Age at Presentation: *COL4A1* Patients

Perinatal and early childhood patients accounted for most *COL4A1* patients, with 17/33 (51%) and 9/33 (27%) falling within these categories, respectively. Table 1 and eTable 2a provide detailed summaries of group-level and individual patient phenotypes, respectively. Medical interventions are detailed in eTable 3. Birth history data are summarized in eTable 4 for the perinatal and early childhood subgroups.

**Table 1:** Clinical Presentation by Age of Presentation *COL4A1* (Total n = 31)

	Perinatal, n (%) n = 17	Early childhood, n (%) n = 9	Late childhood ,n (%) n = 5
Ocular abnormalities^a^	7 (41)	—	2 (67)
Cataracts	5 (29)	—	1 (50)
Glaucoma	2 (12)	—	—
Microphthalmia	1 (6)	—	—
Anterior segment dysgenesis	2 (12)	—	—
Retinal artery tortuosity	—	—	1 (50)
Hearing loss	—	—	2 (40)
Muscle cramps	—	2 (22)	2 (40)
Elevated CK^a^	1 (14)	—	1 (33)
Renal abnormalities^a^	5 (31)	3 (38)	2 (40)
Cardiac abnormalities^a^	2 (18)	—	1 (50)
Feeding difficulties	9 (53)	1 (11)	—
Developmental delays	15 (88)	9 (100)	1 (20)
Global developmental delay	11 (65)	6 (67)	—
Language delays	12 (71)	7 (78)	1 (20)
Motor delays	14 (82)	8 (89)	—
Cerebral palsy	11 (65)	6 (67)	—
GMFCS ≥4	7 (41)	7 (78)	—
Autism	2 (12)	3 (33)	—
ADHD	1 (6)	3 (33)	1 (20)
Learning disability	1 (6)	2 (22)	1 (20)
Intellectual disability	3 (18)	2 (22)	—
Migraines	1 (6)	—	2 (40)
Epilepsy^b^	8 (47)	5 (56)	1 (20)
Infantile spasms	3 (38)	4 (80)	—
Treatment refractory	6 (75)	4 (80)	1 (100)
LGS	1 (13)	3 (60)	—
DEE-SWAS	1 (13)	—	—
Age at epilepsy diagnosis, median (IQR) in years	1.2 (0.8–1.8)	0.6 (0.3–0.7)	5.0
Died	2 (11)	0 (0)	0 (0)

Abbreviations: ADHD = attention deficit hyperactivity disorder; CK = creatine kinase; DEE-SWAS = developmental encephalopathy with spike wave activation in sleep; GMFCS = gross motor function classification system; LGS = Lennox-Gastaut syndrome.

Counts and percentages are presented for categorical variables and median with interquartile range (IQR) for continuous variables. Note that 2 patients are excluded as they were asymptomatic during follow-up.

a Not all patients were screened for specific systemic manifestations; thus, percentage is relative to number of patients undergoing relevant screening rather than the full cohort.

b Percentage for specific epilepsy features is relative to patients with epilepsy only.

### Perinatal *COL4A1* Patients

**Figure 1:**
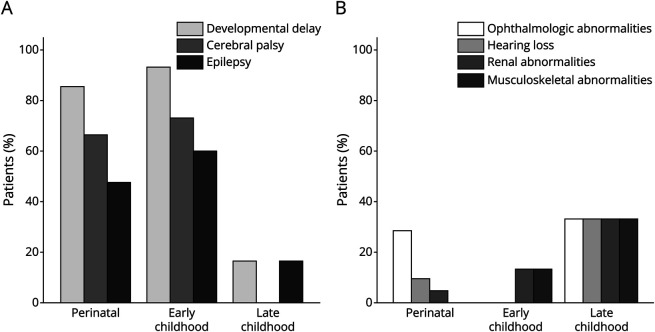
Neurologic and Extra-Neurologic Features by Age at Onset (A) Neurologic features and (B) extra-neurologic features. Percentage of patients is presented by subgroup across both genes (n = 44).

Perinatal patients (n = 17) were followed for a median of 3.3 years (IQR 1.2–4.9). Of these, 9/17 (53%) had abnormal prenatal imaging at a median gestational age of 29 weeks (IQR 22–34). Developmental delays were seen in most patients (15/17, 88%) and were frequently global (11/17, 65%). CP diagnosis was common (11/17, 65%) and frequently severe (7/17 with GMFCS ≥4, 41%) (Figure 1). Autism, ADHD, learning, and intellectual disabilities were diagnosed in a minority, noting that many patients were too young or delayed for such evaluations. Epilepsy was seen in 8/17 (47%) and treatment refractory in most (6/8, 75%). Median age at epilepsy onset was 1.2 years (IQR 0.8–1.8) with infantile spasms in 3/8 patients (38%). Acute symptomatic focal seizures were seen in the perinatal period in 2 patients: one patient then remained seizure free at 5 years while the other developed Lennox-Gastaut syndrome in early toddlerhood.

Regarding extra-CNS findings, all patients underwent ophthalmologic evaluation, with abnormalities seen in 7/17 (41%), including cataracts, glaucoma, microphthalmia, and anterior segment dysgenesis (Figure 1). Renal abnormalities were noted in 5 patients (5/16 only screened, 31%). Screening for cardiac abnormalities or elevated creatine kinase was infrequent; these disease manifestations were uncommon in those screened. Feeding difficulties, conversely, were common (9/17, 53%) with frequent placement of surgical feeding tubes (6/17, 35%). Three patients underwent neurosurgical interventions, namely epilepsy surgery with vagal nerve stimulator placement in one and ventriculoperitoneal shunting in 2 (2/17, 12%). Posthemorrhagic hydrocephalus was uncommon (1/17, 6%).

### Early Childhood *COL4A1* Patients

Early childhood patients (n = 9) presented at a median age of 0.3 years (IQR 0.3–0.9) and were followed for a median of 3.0 years (IQR 0.2–4.6). All patients had developmental delays (100%) with global delays and CP again in most (6/9, 67% and 6/9, 67%), typically severe (7/9 with GMFCS ≥4, 78%). Autism, ADHD, learning, and intellectual disabilities were diagnosed in nearly a third of patients, noting again limitations in assessment given age and delays. Epilepsy was common, affecting 5/9 children (56%), and refractory in most (4/5, 80%). Notably, the one patient without refractory epilepsy was able to wean off antiseizure medication and remained seizure free for 10 years at last follow-up. Nonmedication interventions were pursued in 3/5 (60%), including ketogenic diet (3/5, 60%) and epilepsy surgery (3/5, 60%).

Regarding extra-CNS findings, ophthalmologic evaluation was completed in most children (7/9, 78%), without structural defects. Screening for renal abnormalities was abnormal in 3 patients (3/8, 38%). Cardiac screening was completed in 3/9 patients (33%) without abnormal findings.

### Late Childhood *COL4A1* Patients

Late childhood patients (n = 5) presented at a median age of 10.0 (IQR 6.3–11.0) and were followed for a median of 0.1 years (IQR 0.1–0.1). Developmental delays were infrequent; one child was diagnosed with ADHD, and one had learning difficulties. Two children had migraines (2/5, 40%) and one had epilepsy (1/5, 20%). By contrast, extra-CNS manifestations were common and frequently the symptom leading to evaluation (4/5, 80%), despite low uptake of screening for complications otherwise. Renal abnormalities were the most common manifestation (2/5, 40%). Notably, 2 siblings presented with sensorineural hearing loss; one also had hematuria, proteinuria, muscle cramps, white matter abnormalities, and a porencephalic cyst, while the other had evidence of a subacute unilateral intraventricular hemorrhage. They inherited a *COL4A1* VUS from their father who experienced renal disease and muscle cramps; no additional screening results were available for him.

### Phenotypes by Age at Presentation: *COL4A2* Patients

Perinatal and early childhood patients accounted for most *COL4A2* patients, with only one patient falling into the late childhood group. Table 2 and eTable 2b summarize group and individual phenotypes, respectively. Medical interventions are detailed in eTable 3.

**Table 2:** Clinical Presentation by Age of Presentation *COL4A2* (Total n = 10)

	Perinatal, n (%) n = 3	Early childhood, n (%) n = 6	Late childhood, n (%) n = 1
Ocular abnormalities Retinal hemorrhage	1 (33) 1 (33)	—	—
Renal abnormalities	1 (33)	—	—
Cardiac abnormalities^a^	—	1 (50)	—
Feeding difficulties	1 (33)	1 (17)	—
Developmental delays	3 (100)	5 (83)	—
Global developmental delay	2 (67)	4 (67)	—
Language delay	2 (67)	4 (67)	
Motor delay	3 (100)	4 (67)	
Cerebral palsy GMFCS ≥4	3 (100) 2 (67)	5 (83) 1 (17)	—
Autism	—	—	—
ADHD	1 (33)	1 (17))	—
Learning disability	—	—	—
Intellectual disability	1 (33)	—	—
Migraines	—	1 (17)	+
Epilepsy^b^	2 (66)	4 (67)	—
Infantile spasms	1 (50)	1 (25)	—
Treatment refractory	—	2 (50)	—
LGS	—	—	—
Age at epilepsy diagnosis, median (IQR) in years	0.4 (0.0–0.8)	1.7 (0.8–2.5)	—

Abbreviations: ADHD = attention deficit hyperactivity disorder; GMFCS = gross motor function classification system; LGS = Lennox-Gastaut syndrome.

Counts and percentages are presented for categorical variables and median with interquartile range (IQR) for continuous variables.

a Not all patients were screened for specific systemic manifestations; thus, percentage is relative to number of patients undergoing relevant screening rather than the full cohort.

b Percentage for specific epilepsy features is relative to patients with epilepsy only.

Perinatal patients (n = 3) were followed for a median of 0.9 years (IQR 0.4–1.7). Early childhood patients (n = 6) presented and were followed for a median age of 0.5 years (IQR 0.4–1.3) and 1.3 years (IQR, 0.6–1.8 years), respectively. Developmental delays were appreciated in all perinatal and most early childhood patients (3/3, 100%, and 5/6, 83%). Most experienced global developmental delays (2/3 perinatal, 67%, and 4/6 early childhood, 67%, respectively) and were diagnosed with CP (3/3 perinatal, 100%, and 5/6 early childhood, 83%, respectively) (Figure 1). Two children were diagnosed with ADHD. Epilepsy was seen in two-thirds of children (2/3 perinatal, 67%, and 4/6 early childhood, 67%, respectively). The median age at epilepsy onset was 0.4 (IQR 0.0–0.8) and 1.7 years (IQR 0.8–2.5) for the perinatal and early childhood groups. One perinatal patient underwent endoscopic third ventriculostomy for suspected congenital aqueductal stenosis.

Extra-neurologic manifestations were infrequent (Figure 1). Retinal hemorrhage was noted in one perinatal onset patient. Screening for renal complications was completed for most patients and largely unrevealing. Cardiac screening, performed in all perinatal and a third of early childhood onset patients, was similarly negative for most.

### Neuroimaging

#### *COL4A1* Cohort

**Figure 2:**
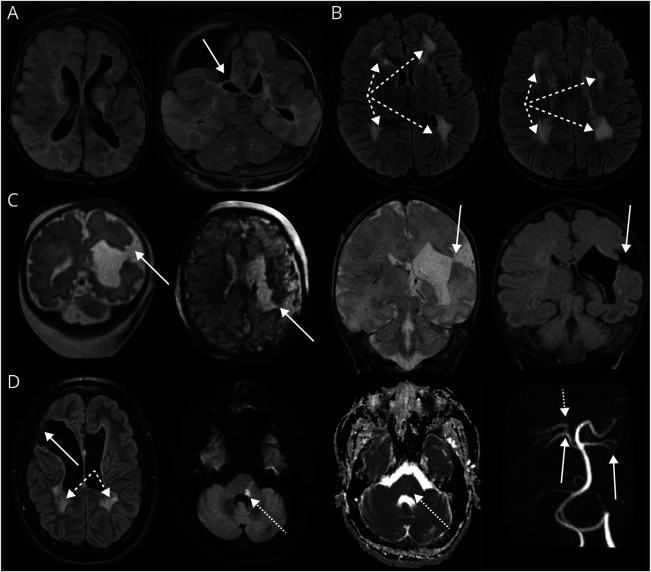
Neuroimaging Patterns in *COL4A1*-Related Disorder (A) PVHI pattern with porencephalic cysts (thick white arrow) in a patient with *COL4A1* variant with perinatal presentation (axial and coronal T2/FLAIR), (B) SVID pattern (dashed thick arrows) in patient with *COL4A1* variant with late childhood presentation (axial T2/FLAIR), (C) PVHI pattern (thick white arrows) in patient with *COL4A1* variant, fetal MRI (coronal HASTE and axial EPI SWI), and postnatal MRI (coronal T2WI and T1WI), (D) PVHI pattern (thick white arrows) on SVID background (dashed thick arrows) in a patient with *COL4A1* variant with perinatal presentation despite diagnosis being delayed until late adolescence secondary to a subacute left pontine stroke shown on DWI/ADC (dotted thick arrows). Vessel imaging demonstrates tortuosity of posterior circulation, particularly involving the vertebrobasilar system with dominant left vertebral artery and irregularity of right posterior cerebral (thin dotted arrow) and bilateral superior cerebellar arteries (thin white arrows) on MR angiogram. Each panel represents a different patient. ADC = apparent diffusion coefficient; DWI = diffusion-weighted imaging; EPI SWI = echo planar imaging susceptibility weighted imaging; FLAIR = fluid-attenuated inversion recovery; HASTE = half Fourier acquisition single-shot turbo spin echo; PVHI = periventricular hemorrhagic infarction; SVID = small vessel ischemic disease; T1WI = T1-weighted imaging; T2WI = T2-weighted imaging.

Original neuroimaging was available in 14 perinatal (14/17, 82%), 8 early childhood (8/9, 89%), and all late childhood patients (5/5, 100%). Figure 2 shows representative images, and eTable 5 summarizes key neuroimaging findings across the study population.

Overall, most perinatal patients had an imaging pattern consistent with periventricular hemorrhagic infarction (14/17, 83%) (PVHI), which was superimposed in 4 on a background of leukoencephalopathy/small vessel ischemic disease (SVID). Schizencephaly and porencephaly were evident in some (4/17, 24%, and 9/17, 53%, respectively). One patient had extensive brain malformations consistent with hydranencephaly spectrum (1/17, 6%). Posterior fossa malformations were noted in 6/17 patients (35%). Ventriculomegaly was common (13/17, 76%) with posthemorrhagic hydrocephalus in 3 patients (3/17, 18%). Intracranial vessel tortuosity was noted in 4/17 (24%) of patients, noting dedicated vessel imaging was infrequent. Median neuroimaging severity score was 22 (IQR 13–31) and severe in 10/14 patients (71%).

In the early childhood patients, PVHI was observed in most (6/9, 67%) and superimposed on an SVID background in all. Porencephaly was common (6/9, 67%), as were posterior fossa malformations, ventriculomegaly, and intracranial vessel tortuosity (4/9 (44%), 7/9 (78%), and 5/9 (56%) patients, respectively). One patient with global developmental delays and renal disease had vessel tortuosity only. Median neuroimaging severity score was 20 (IQR 10–28) and severe in 6/9 patients (67%).

All late childhood patients had an SVID background with diffuse white matter signal abnormality reminiscent of leukodystrophies. One patient had intracranial vessel tortuosity. Median imaging severity score was 8 (IQR 4–9), with no severe scores.

#### *COL4A2* Cohort

**Figure 3:**
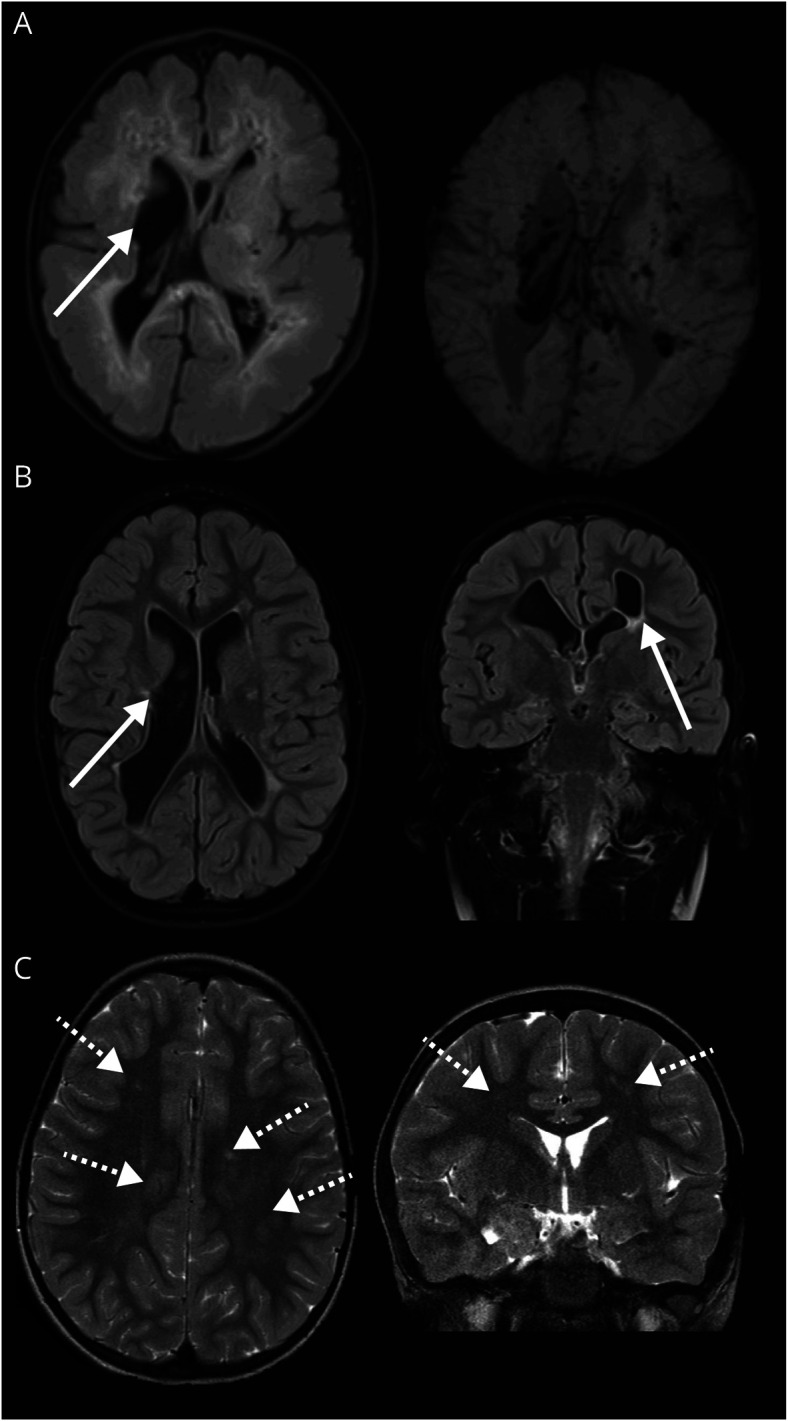
Neuroimaging Patterns in *COL4A2*-Related Disorder (A) Porencephaly with basal ganglia involvement and PVHI pattern (thick white arrow) in a patient with *COL4A2* variant with perinatal presentation, imaged in first 2 weeks of life (axial T2/FLAIR, GRE), (B) porencephaly with basal ganglia involvement and PVHI pattern (thick white arrows) in patient with *COL4A2* variant with early childhood presentation (axial and coronal T2/FLAIR), and (C) nonspecific small foci of hyperintense signal on T2WI in subcortical white matter in a patient with *COL4A2* variant with late childhood presentation (axial and coronal T2) (dashed thick arrows). Each panel represents a different patient. FLAIR = fluid-attenuated inversion recovery; GRE = gradient echo; PVHI = periventricular hemorrhagic infarction; T2WI = T2-weighted imaging.

In the *COL4A2* cohort, neuroimaging was available in all perinatal and 4 early childhood patients. Figure 3 shows representative images. Perinatal and early childhood patients again typically featured a PVHI pattern, often on an SVID background, and with porencephaly in all (2/3, 67%, and 4/6, 67%). One early childhood patient, whose imaging was not available for review, reportedly had normal imaging; vessel imaging was not completed. Posterior fossa malformations were seen in two-thirds of perinatal patients (2/3, 67%). Median neuroimaging severity scores were 22 (IQR 3–27) and 19 (IQR 14–24) for perinatal and early childhood patients, respectively. Scores fell within the severe range in 2 (2/3, 67%) and 3 patients (3/6, 50%), respectively.

### Genetics

**Figure 4:**
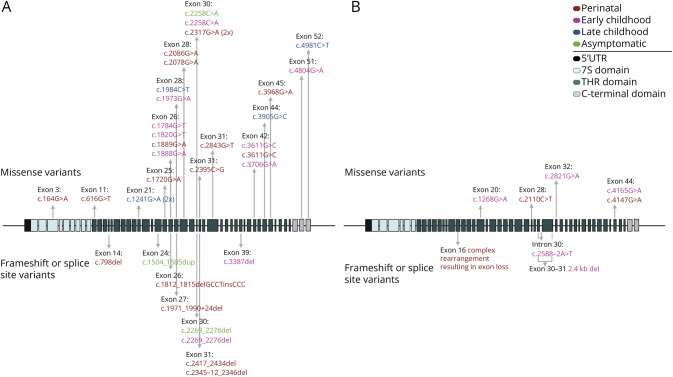
Genetic Variants (A) *COL4A1* (NM_001845.6) and (B) *COL4A2* (NM_001846.4). Top panels reflect missense variants, while lower include frameshift and splice site variants. Variants are labeled based on age at disease onset. Variants noted in multiple patients are followed by the number of affected patients (*n* x) unless phenotype varies across individual patients, in which case each patient is listed separately.

Genetic testing reports were available for all but 2 patients; these had *COL4A2* variants reported to be disease-causing by an international laboratory >2 decades ago. Otherwise, there were 30 and 9 distinct variants in *COL4A1* and *COL4A2,* respectively. Four of these were seen in 2 patients each, including 3 sets of siblings, notably with phenotypic variability in one set (asymptomatic into school-aged years compared with early onset CP, epilepsy, and renal disease). This calculation also accounts for the patient with variants in both genes. As previously noted in the literature, most variants were located in the triple helix region. Overall, we identified several variants not previously reported or submitted to ClinVar. No genotype-phenotype correlations were apparent. Variant location and type are depicted in Figure 4, and variant details are included in eTable 2a–c.

### Neuroimaging Over Time

Prenatal and postnatal imaging were available for 5 patients with *COL4A1* variants. Expected evolution of injuries was common, while accrual of new lesions was not. Twelve *COL4A1* patients underwent interval postnatal imaging, with the last image acquired at a median of 2.0 years (IQR 1.6–5.0). Disease-related interval changes were noted in 3 patients and included progression of white matter gliosis, new parenchymal microhemorrhages, and a subacute pontine stroke in late adolescence in a patient with perinatal onset disease. In this case, a thorough evaluation for stroke risk factors was pursued and unrevealing (eTable 2a for details). *COL4A2* patients underwent interval postnatal imaging with a median interscan time of 0.8 years (IQR 0.04–2.1). Interval changes were noted in one child with progressive diffuse white matter gliosis. The one child with variants in both genes underwent interval reimaging in the setting of multiorgan failure; significant progression of white matter injury was evident, with new calcifications, cortical necrosis, cerebellar and parenchymal microhemorrhages, and overall volume loss. Overall, no large hemorrhages were observed on interval reimaging.

### Associations With Profound Neurologic Impairment

**Figure 5:**
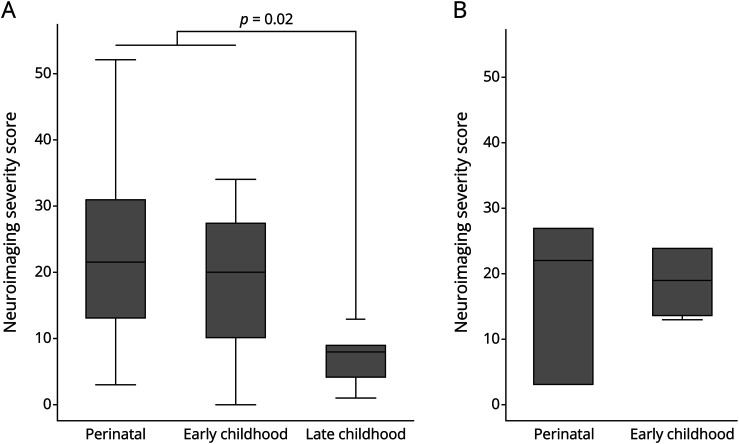
Neuroimaging Severity Score by Age at Presentation Category (A) *COL4A1* and (B) *COL4A2*. Note, original images were not available for review in the one late childhood *COL4A2* patient. Severity scores were significantly higher when comparing the late childhood *COL4A1* group with both perinatal and early childhood groups combined.

There was no difference in the proportion of patients with profound neurologic impairment by gene (*p* = 0.7). There was a difference, however, in the proportion of patients with profound impairment across age-based phenotype categories (*p* = 0.009), with 80% of perinatal and early childhood patients (16/20 and 12/15, respectively) experiencing such an outcome, compared with 17% of late childhood patients (1/6). Imaging scores were frequently elevated in the *COL4A1* perinatal and early childhood groups; when compared with the late childhood group, this difference was significant (*p* = 0.02) (Figure 5). Such a comparison was not possible for *COL4A2* given sample size.

On univariable logistic regression, age at presentation was associated with profound neurologic impairment, with a 38% reduction in odds of experiencing profound impairment for every year increase in age (OR 0.6, 95% CI 0.4–1.0, *p* = 0.03). Imaging severity was also associated with impairment, with 10% increased odds of profound impairment for every severity point (OR 1.1, 95% CI 1.0–1.2, *p* = 0.02, score taken as continuous) and a 9-fold increase in odds in children with a severe score (OR 9.0, 95% CI 1.8–44.6, *p* = 0.007). These associations persisted in sensitivity analyses excluding motor function from the definition of profound neurologic impairment. This effect was not confounded by sex or gene (<10% in effect estimate), nor did these covariates improve model fit. Age at presentation and imaging severity score were not included in the multivariable model given expected causal association.

## Discussion

We present 44 pediatric patients with *COL4A1* and *COL4A2*-related disorder evaluated at a large institution and identified through an unbiased systematic search of its EHR. We aimed to identify subgroups with distinct clinical and neuroimaging features, hypothesizing that early onset correlates with severe manifestations. We identified 3 subgroups based on age at onset: 1) perinatal, recognized from prenatal to 28 days old; 2) early childhood, from postperinatal to 4 years old; and 3) late childhood, from 4 to 18 years old, comprising nearly half, one-third, and one-quarter of the sample, respectively. Notably, perinatal and early childhood patients share many similarities in phenotype and neuroimaging. It is possible that at least some early childhood patients could have been recognized prenatally had they undergone fetal neuroimaging. These 2 subgroups may converge with future advances in prenatal evaluation. Conversely, early childhood cases may also reflect a spectrum of severity with earlier onset patients sharing greater overlap with perinatal patients and boundaries that will evolve with future studies. Our sample size did not allow for more systematic approaches to subgroup identification such as latent class analysis, which would be worthwhile to pursue with consolidated cohorts across institutions. This classification system, however, is clinically relevant in the absence of routine advanced fetal neuroimaging.

Patients with perinatal and early childhood onset disease had a prominent neurologic phenotype with overlapping features across disorders. Most children had developmental delays, complicated by CP in at least two-thirds and epilepsy in approximately half. Conversely, late childhood presentations had less severe phenotypes, with infrequent developmental delays, CP, and epilepsy. This spectrum of neurologic manifestations is consistent with the literature, although the prevalence of specific manifestations varies across reports.^1-21^ In a study focused on the neurologic manifestations of *COL4A1* and *COL4A2*-related disorder, epilepsy was documented in most patients (55/123, 45%, and 38/44, 86%, respectively).^11^ Although most patients also had other neurologic features, a subgroup was recognized with isolated epilepsy as well. In a more recent review of over 600 published patients, neurologic manifestations were similarly noted in nearly two-thirds of cases (507/647, 78%).^20^ Manifestations included epilepsy, intellectual disability/developmental delay, and motor deficits, each seen in approximately one-fifth to one-quarter of patients. Notably, caregivers who completed a tailored questionnaire reported a history of developmental delays in a proportion of adult patients, which highlights the need to differentiate between age at disease onset and diagnosis. We suspect that discrepancies in estimates across studies are likely because of varying inclusion of disease subgroups.

Extra-neurologic manifestations varied by subgroup and disorder. *COL4A1* variants are associated with ophthalmologic disease, including congenital cataracts, microphthalmia, anterior segment dysgenesis, and retinal arterial tortuosity, which often lead to glaucoma, hemorrhage, retinal detachment, and optic neuropathy.^21^ Similar features are less commonly seen with *COL4A2* variants.^2,20,28^ In our study, ophthalmologic manifestations were frequent in perinatal and late childhood patients with *COL4A1*, less so with *COL4A2*. Renal abnormalities and muscle cramping were more frequent in late childhood patients, aligning with the HANAC phenotype.^29-32^ Cardiac abnormalities were infrequent. Notably, we identified 2 adolescents with sensorineural hearing loss in addition to neuroimaging and renal features typical of *COL4A1/2*-related disorders. To our knowledge, sensorineural hearing loss has not been previously described in this disease, despite being common with variants in *COL4A3-5*, which also lead to ocular and renal disease.^33^ One of these patients, furthermore, had evidence of an asymptomatic porencephalic cyst despite having an overall HANAC phenotype, which is unusual among described patients.^34^

Neuroimaging features varied across the 3 subgroups. Perinatal and early childhood patients often showed periventricular hemorrhagic infarction. Such injury is a common neurologic complication of prematurity with decreasing incidence with older gestational age (unusual after 32 weeks gestation).^35-37^ In *COL4A1/2*-related disease, injury may occur both prenatally and perinatally secondary to fragile vascular endothelium, leading to gliosis, porencephalic cysts, schizencephaly, cerebellar abnormalities, and neuronal migrational abnormalities, depending on the timing of the insult. In some patients, PVHI was superimposed on a background of white matter injury, akin to changes seen in SVID. Cystic lesions were common. Late childhood presentations, conversely, exhibited patchy white matter signal change typically without cystic lesions. This spectrum of neuroimaging findings with variation across the lifespan is reflected in the literature.^4,7,18,38^ Perinatal and early childhood presentations typically had more severe injuries on imaging compared with late childhood presentations, as assessed through an adapted severity score.^26^ Similarly, the proportion of patients experiencing profound neurologic impairment varied from most perinatal and early childhood onset cases to the minority of late-onset patients. The odds of experiencing profound impairment decreased with increasing age at onset and increased with worsening neuroimaging severity score.

Serial imaging in a subset of patients with a median time to last image of approximately 2 years suggests that accrual of new injury is infrequent. Most notable in our cohort was a patient with perinatal onset *COL4A1*-related disease who experienced a pontine ischemic stroke in adolescence. Thus, while serial imaging in early childhood may be low yield, it may have a role in adolescence and adulthood.^22^

Limitations to our study include its retrospective design, limited follow-up, and relatively small sample size. Despite access to detailed medical records, retrospective data collection resulted in a degree of missingness because of inconsistent reporting of features of interest and screening studies. This approach may have resulted in measurement bias, with lower rates of disease features, particularly non-neurologic, within this cohort, and limited ability to identify rarer disease complications given sample size as well. Selection bias is also possible, in that patients with more severe manifestations may both be more likely to undergo diagnostic testing and thus be identified, and then to follow closely with their medical teams with further evaluations. The result would be overestimates of overall disease severity and complications, as milder presentations would be missed. Of note, we do not report on indications for testing on a case-by-case basis as testing was pursued by multiple providers across disciplines with varying thoroughness in reported phenotypes and indications. We did, however, include 2 asymptomatic patients who underwent testing given a symptomatic parent. Limited follow-up may have hampered our ability to identify evolution of phenotype over time in these as well as other patients, as well as cognitive and behavioral outcomes such as autism, learning or intellectual disabilities in older children. Finally, while we systematically reviewed available imaging to assess severity of injury using a score developed for a pediatric population with overlapping imaging features, this severity score has not been specifically validated in this population. Developing such a validated score was beyond the scope of this project, but worth pursuing as long-term sequelae of injury, such as extent of cystic and cortical malformations, may be incompletely captured by this tool.

Despite these limitations, our study contributes to the literature by providing in-depth phenotyping on a sizable cohort of new patients, with a median follow-up time of 1–3 years. While not systematically pursued, screening for non-neurologic manifestations was completed in most patients, reflecting the growing awareness of these disorders' complexity and the need for multidisciplinary care.^20,22^ Another strength is our unbiased approach to patient identification through an EHR-based query, which enabled us to identify patients presenting across the institution without the selection bias that would have been introduced by collecting patients from a single subspecialty clinic. Finally, centralized review of neuroimaging enabled the consistent application of a score to estimate severity, a tool that may be used in further studies of longitudinal imaging to better understand the extent of disease progression and its prognostic value.

*COL4A1/2*-related disorder has a broad phenotypic spectrum with multisystem involvement and disease onset ranging from fetal life into adulthood. Among pediatric patients, disease subgroups with distinct clinical and neuroimaging features can be identified. Perinatal and early childhood presentations exhibit a more prominent neurologic phenotype compared with late childhood presentations. Except for ophthalmologic manifestations, late childhood patients more frequently exhibit systemic manifestations such as renal and musculoskeletal involvement. Imaging ranges from patterns of extensive early injury consistent with periventricular hemorrhagic infarction to leukoencephalopathy in early and late childhood onset disease, respectively. Future work is needed to understand evolution of disease over time. Aided by cohort stratification, such work will be crucial for the development of targeted management guidelines and improved prognostication.

## Glossary

ADHD: attention deficit hyperactivity disorder
CNV: copy number variant
CP: cerebral palsy
GMFCS: gross motor function classification system
HANAC: hereditary angiopathy and nephropathy with aneurysms and muscle cramps
IQR: interquartile range
LGS: Lennox-Gastaut syndrome
